# Results of the Carillon Procedure in Proportionate and Disproportionate FMR: Effects of the Baseline Echocardiographic Parameters

**DOI:** 10.3390/jcm15176641

**Published:** 2026-08-28

**Authors:** Piotr Kałmucki, Rafał Dankowski, Janusz Lipiecki, Artur Baszko, Tomasz Siminiak

**Affiliations:** 1 HCP Medical Center, Poznan University of Medical Sciences, 61-485 Poznan, Poland; rafaldan@ump.edu.pl (R.D.); abaszko@ump.edu.pl (A.B.); tsiminia@ump.edu.pl (T.S.); 2 Clinique Pôle Santé République, 63-050 Clermont Ferrand, France; jlipiecki@polesanterepublique.com

**Keywords:** mitral regurgitation, mitral annuloplasty, Carillon device

## Abstract

**Background:** Transcatheter edge-to-edge repair and percutaneous mitral annuloplasty with the Carillon device are techniques used in the treatment of functional mitral regurgitation (FMR). Both approaches have been shown to have divergent clinical outcomes in patients with proportionately severe and disproportionately severe FMR. **Objective:** To evaluate which patients could benefit most from percutaneous mitral annuloplasty, we evaluated the possible correlation between echocardiographic parameters at baseline and their change one year after the Carillon procedure in subjects with proportionate versus disproportionate FMR. **Methods:** We performed a pooled analysis from three trials of patients with FMR. The main inclusion criteria included persistent grades 2+ to 4+ FMR with >5.5 cm left ventricular (LV) end-diastolic diameters (LVEDDs) and a reduced LV ejection fraction. Patients with an effective regurgitant orifice area/LV end-diastolic volume (EROA/LVEDV) ratio equal to 0.15 mm^2^/mL or less were assigned to the proportionate FMR group (n = 74; 65%), and those with a ratio above 0.15 mm^2^/mL were classified as having disproportionate FMR (n = 39; 35%). **Results:** In both groups, the changes in LV systolic and diastolic volumes after the procedure correlate negatively with baseline LV volumes, suggesting larger improvements (decreases) in LV volumes in patients with more pronounced LV dilatation at the baseline. In contrast to the disproportionate group, in patients with proportionate FMR, no correlation between the baseline LV diameters and improvement in LV volumes after 12 months was observed. However, higher baseline LV diameters predicted a higher degree of decrease in LV diameters in “proportionate” but not “disproportionate” FMR patients. Interestingly, the improvement in EF as a result of the Carillon implantation was not predicted by any of the baseline parameters studied. However, in both groups, the 12-month change in mitral annular area negatively correlated with baseline vena contracta but not with RV and EROA. **Conclusions:** In FMR patients undergoing the Carillon procedure, higher baseline LV volumes may predict more pronounced reverse remodeling, defined as a decrease in LV volumes. Higher values of vena contracta may predict larger reductions in the mitral annular area. High baseline LV diameters could suggest improvements in LV volumes in disproportionate FMR and in LV diameters in proportionate FMR, probably due to different LV geometries.

## 1. Introduction

Functional mitral regurgitation (FMR) in patients with heart failure (HF) is very common and associated with a poor prognosis [1]. Due to the high risk of cardiac surgical procedures, especially in patients with reduced left ventricular (LV) ejection fractions (EFs), percutaneous procedures to treat FMR have been implemented into clinical practice. These include transcatheter edge-to-edge repair (TEER) techniques targeting mitral leaflets, and a percutaneous transvenous mitral annuloplasty technique that reduces mitral annulus dimensions, leading to an improvement in mitral valvular coaptation [2,3]. This latter technique, which utilizes the Carillon Mitral Contour System (CMCS), is the only treatment approach that does not lead to irreversible damage of the mitral apparatus, enabling options for all types of future interventions, if needed [4].

The clinical benefit of the Carillon procedure in patients with FMR has been shown in several multicenter randomized trials demonstrating improvement in echocardiographic parameters of FMR, as well as in reverse LV remodeling, defined as a decrease in LV diameters and volumes [5,6,7]. The results of these clinical trials have shown consistent clinical benefit in heart failure patients with FMR; however, as with all other treatments techniques, outcomes in individual patients may vary. Understanding which subjects could benefit the most from the CMCS is important in patient selection. Therefore, we thought to analyze possible correlations between the baseline echocardiographic parameters and the improvements after the procedure.

The divergent clinical outcomes of two major clinical trials evaluating TEER in patients with FMR—the COAPT and MITRA-FR trials—as well as the differences in their inclusion criteria [2,3], has led to the concept of “proportionate” and “disproportionate” FMR based on the relationship between the severity of MR and the degree of LV dilatation [8,9]. Although this concept has not yet been universally accepted, current clinical guidelines recommend using TEER in COAPT-like patients, i.e., with disproportionate FMR, presenting the degree of MR greater than expected by the degree of LV dilatation [10].

Our previously published analysis has also indicated a difference in the results of the Carillon procedure in patients with proportionate and disproportionate FMR. Percutaneous annuloplasty using this technique resulted in improvement of echocardiographic parameters in both groups of patients, but LV reverse remodeling was more apparent in patients with proportionate FMR [11].

To verify which individual patients may benefit the most from the percutaneous mitral valvuloplasty with the Carillon system, we performed an analysis of correlations between the baseline echocardiographic parameters of FMR and the degree of changes in both LV sizes and volumes, as well as in MR parameters, one year after the procedure.

## 2. Methods

We performed a pooled analysis of patients with FMR treated with a percutaneous mitral annuloplasty using CMCS in multicenter clinical trials: TITAN [5], TITAN II [6], and REDUCE FMR [7] trials. Ethics committees at each site approved the protocol of each trial, and all subjects provided written informed consent. Patients met previously published inclusion and exclusion criteria [5,6,7], including ongoing symptomatic HF and grades 2+ to 4+ FMR despite guideline-directed HF medication, with >5.5 cm LV end-diastolic diameters (LVEDDs), and reduced LV ejection fractions (<40% in TITAN and TITAN II, <50% in REDUCE-FMR). The main exclusion criteria were: surgical or percutaneous coronary revascularization within the previous 30 days, severe mitral valve degenerative pathology or mitral annular calcification, or previous mitral valve surgery, as well as a current or indicated cardiac resynchronization device. Blinded independent core echocardiography laboratory analysis was undertaken at study termination. MR grading was based upon American Society of Echocardiography recommendations [12].

The CMCS procedure has been described previously [13]. The procedure takes advantage of the anatomical proximity of the coronary sinus to the mitral annulus for annular reduction. Briefly, the Carillon (Kirkland, WA, USA) device is a nitinol fixed-length double-anchor device. The distal anchor is deployed distally in the coronary sinus. Traction of the system allows for the application of tension to plicate the posterior mitral annulus. A proximal anchor is then deployed near the coronary sinus ostium to maintain the tension.

All patients underwent independent core laboratories echocardiographic parameter evaluations, which included MR regurgitant volume (RV), effective regurgitant orifice area (EROA), vena contracta (VC), LV ejection fraction (LVEF), LV end-diastolic volume (LVEDV), LV end-systolic volume (LVESV), LV end-diastolic diameter (LVEDD), LV end-systolic diameter (LVESD), and mitral annulus area (MAA).

As previously described, patients with baseline effective regurgitant orifice area (EROA)/LV end-diastolic volume (LVEDV) ratios of 0.15 or less were assigned to the proportionate FMR group, and those with a ratio above 0.15 were classed as having disproportionate FMR [14].

### Statistical Analysis

All data were taken at baseline before the procedure and after a one-year follow-up. Continuous variables are shown as means and standard deviations for normally distributed data, or medians and interquartile ranges for non-parametric data. Categorical variables are presented as counts and percentages. Changes in echocardiographic variables over 1 year relative to the baseline were analyzed with a paired samples *t*-test.

Comparison of LV and mitral valve changes in proportionate FMR and disproportionate FMR groups were analyzed using analysis of covariance adjusted for baseline values. Pearson’s correlation coefficient (for normally distributed) or Spearman’s rank correlation coefficient (for non-normally distributed data) was reported to assess the relationship between echocardiographic parameters at baseline and changes in echocardiographic parameters at the 1-year follow-up. Statistical significance was defined as *p* < 0.05. All tests were two-sided but should be considered in the context of this retrospectiv post hoc setting, while no controls for multiplicity of testing were used. Statistical analyses were performed by an independent biostatistician using Stata v16 (StataCorp, College Station, TX, USA).

## 3. Results

Across the three studies pooled for the current analysis, 139 patients were treated with the Carillon device, and the baseline characteristics of these patients have been previously reported [11]. Overall, 113 of these patients, aged 67 ± 11 years, with complete echocardiographic datasets at baseline and 12 months, were included in this analysis. A total of 74 (65%) patients were classified as presenting proportionate FMR, whereas 39 (35%) presented with disproportionate FMR. Patients with proportionate FMR were younger (65 ± 12 vs. 72 ± 7 years, *p* < 0.05) and, by the group’s definition, had greater LV dilatation (<0.01 for all LV diameter and volume measures). Similarly, proportionate FMR patients showed lower baseline LV ejection fractions (29 ± 8 vs. 34 ± 8%, *p* = 0.005), although the forward stroke volume was comparable between groups [11]. Patients in both groups had similar percentages of ischemic etiologies of LV dilatation (69% vs. 71%) and histories of myocardial infarction (53% vs. 54%, for proportionate and disproportionate FMR, respectively). There were no significant differences in concomitant morbidity, including diabetes mellitus (22% vs. 21%, *p* > 0.99) and atrial fibrillation (45% vs. 62%, *p* = 0.11). Both groups at baseline had similar left atrial volumes (101 ± 40 vs. 109 ± 49 cc; *p* = 0.34) and NT-proBNP levels (2987 ± 2657 vs. 4003 ± 3560 pg/mL; *p* = 0.26).

The patients were required to adhere to the HF guidelines’ medication throughout the whole duration of the study. There was no statistical difference in mortality after one year between the groups studied (15.3% vs. 25.6, log-rank *p* = 0.16).

The effects of percutaneous mitral annuloplasty with CMCS in both proportionate and disproportionate patients has been shown in our previous study [11]. The procedure reduced mitral regurgitation in patients with both proportionate and disproportionate FMR: at 12 months following the treatment, both groups showed improvements in regurgitation volume, EROA, and vena contracta. In patients with proportionate FMR, clinically relevant and statistically significant improvements in LV volumes and diameters were observed, whereas this effect did not reach statistical significance in subjects with disproportionate FMR, indicating that in patients with proportionate FMR, the procedure is more likely to result in reverse LV remodeling [11]. However, there was no independent relationship between the degree of proportionality as a continuous variable and the remodeling response to the therapy [11].

Since, as in every treatment modality, the individual response to percutaneous mitral annuloplasty varies between subjects substantially, we analyzed the possible relationships between the baseline patients’ characteristics and the changes in echocardiographic parameters of mitral regurgitation in combination with LV remodeling. The exact values of Spearman’s or Pearsons’s rank correlation coefficients, together with statistical significance levels, are summarized in Table 1 and Table 2.

In both groups, the changes in both left ventricular end-systolic volumes (LVESVs) and end-diastolic volumes (LVEDVs) after the procedure correlated negatively with baseline LV volumes. Since changes in LV volumes after the Carillon procedure had negative values (LV volumes decreasing), this negative correlation is suggestive of greater improvements (decrease) in LV volumes in patients with more pronounced LV dilatation at baseline in both groups studied.

In contrast to the disproportionate (Table 1) group, in patients with proportionate FMR (Table 2), no correlation between the baseline LV diameters and improvement in LV volumes after 12 months was observed. However, higher baseline LV diameters predicted a higher degree of decrease in LV diameters in proportionate but not disproportionate FMR patients.

Interestingly, the improvement in LVEF as a result of the CMCS implantation was predicted by any of the baseline parameters studied. However, LVEF correlated with LVESV changes in both groups (r = 0.458 and r = 0.483 in proportionate and disproportionate groups, respectively) and with LVEDV in patients with disproportionate FMR (r = 0.394), indicating that a low LVEF at baseline may predict more pronounced reverse remodeling, defined as an increased reduction in LV volume, 12 months after the procedure.

In both groups, the 12-month changes in mitral annular area (MAA) negatively correlated with the baseline vena contracta (r = −0.459 and r = −0.374 for “proportionate” and “disproportionate” groups, respectively) but not with RV and EROA. The differences between the groups studied in the correlations between baseline MR echocardiographic parameters and their changes 12 months after the procedure were observed; for the proportionate FMR group (Table 2), the baseline VC correlated significantly with EROA and VC changes (decreases), whereas in disproportionate FMR patients, a higher baseline EROA predicted a greater reduction in MR regurgitant volume and EROA (Table 1).

## 4. Discussion

Previous reports have indicated that the effects of TEER procedures in patients with FMR and heart failure with reduced EF may be dependent on the patient population studied [15,16]. Patients with FMR greater than expected according to their LV dilatation (disproportionate FMR) were shown to respond to the TEER technique more favorably, as compared to those in whom the degree of FMR is proportionate to LV enlargement. Although the definitions of the proportionality of FMR is not universally accepted, it is frequently used in clinical practice, as the clinical guidelines have reflected this hypothesis and provided a higher recommendation for TEER in patients with disproportionate FMR [10].

We have previously shown in [11] that percutaneous mitral annuloplasty with the Carillon device reduces all key echocardiographic parameters of mitral regurgitation, including vena contracta, EROA, and regurgitant volume, in patients with both proportionate and disproportionate FMR. However, there was also a difference in the response to the procedure between both groups: patients with proportionate FMR presented with a greater reduction in LV volumes and diameters one year after the Carillon procedure, indicating a more pronounced LV reverse remodeling [11].

The clinical benefit from percutaneous mitral annuloplasty has been shown consistently in all clinical trials [5,6,17,18], including improvements in HF symptoms. However, similar to all treatment methods, the degree of improvement between each individual patients may vary substantially. To evaluate the possible value of baseline echocardiographic parameters in the prediction of the 12-month efficacy of the Carillon procedure in patients with proportionate and disproportionate FMR, the possible relationships between the improvements in echocardiographic parameters of FMR and in LV size and function with these parameters at baseline were calculated, from which the data are summarized in Table 1 and Table 2. In both groups, the changes in left ventricular systolic and diastolic volumes correlated negatively with baseline LV volumes. Given that, as a result of the procedure, the changes in LVESV and LVEDV had negative values, such a negative correlation suggests a greater improvement in LV volumes in patients with more pronounced LV dilatations at the baseline. Thus, independently of the proportionality of FMR, the degree of reverse remodeling of the LV (defined as a reduction in LV volume) as a result of Carillon implantation is predicted by the baseline degree of dilatation, as assessed by LV systolic and diastolic volumes. Similarly, a decreased baseline LV ejection fraction may suggest a higher probability of greater reduction in systolic LV volumes in both groups of patients with FMR.

However, both groups studied have shown divergent correlations between the baseline LV diameters and the changes in LV diameters and volumes as a result of percutaneous mitral annuloplasty with the CMCS. In contrast to patients with disproportionate FMR, in the proportionate group, no correlation between the baseline LV diameters and improvement in LV volumes after 12 months was observed (Table 1 and Table 2). Since previous reports have shown significant improvements in patients with “proportionate” FMR, this finding may indicate that, in proportionate FMR patients, relatively smaller LV diameters at baseline do not exclude important decreases in LV volumes 12 months after the procedure. The differences in the predictions of the reductions in LV volumes and diameters by assessment of the baseline LV diameters and volumes are likely to be related to the different geometries of the LV in both groups of patients.

Interestingly, the improvement in EF as a result of the Carillon implantation was not predicted by any of the baseline parameters studied, neither in proportionate nor in disproportionate FMR groups. However, in both groups, 12-month changes in the mitral annular area negatively correlated with baseline VC but not with RV and EROA, indicating that a greater reduction in the mitral annular area has been achieved in subjects with bigger vena contracta values at baseline. In addition, greater baseline vena contracta indicated a higher probability of more pronounced reductions in vena contracta after the procedure in both groups studied, though in proportionate FMR patients, there was also a greater reduction in EROA. The possible causes for the divergent contribution between the baseline VC and baseline EROA in the predictions of the outcomes of procedures between the two groups warrant further analysis.

The results of our study confirm our previous observations indicating a divergent response to mitral annuloplasty with CMCS between patients with proportionate and disproportionate FMR [11]. However, the selection of optimal candidate patients based on baseline echocardiographic parameters of mitral regurgitation and/or LV dilatation remains difficult. In disproportionate FMR, greater reverse LV remodeling (defined as decrease in LV volumes) could be expected in patients presenting at baseline with larger LV volumes and diameters, as well as greater annular areas and more impaired ejection fractions. On the other hand, as previously shown [11,19], patients with proportionate FMR generally have a better response to Carillon therapy as far as LV remodeling is concerned [11]; therefore, those with somewhat lesser LV dilatations may result in substantial LV remodeling—the correlations between baseline dilatation and remodeling after the procedure are less apparent.

It has been shown that patients with recurrent MR after TEER were characterized by a pre-procedural high left ventricular sphericity index and left atrium enlargement [20,21]. A spherical-shaped left ventricle may reflect an advanced state of heart failure, with progressive changes in the ventricular geometry and reduced capability of reverse remodeling [21]. Irrespective of MR, unbalanced left ventricular geometries and shapes have been associated with cardiovascular events and heart failure [22]. Patients with “proportionate” FMR present with a more spherical LV. It could be speculated that the divergent relation between baseline LV diameters and changes in LV volumes 12 months after the CMCS procedures observed in our study is related to the degree of sphericity of the left ventricle.

The concept of FMR proportionality has not been fully accepted and incorporated into the clinical practice despite data showing that, in patients under medical therapy, the degree of FMR disproportionality predicts mortality and its predicative power is higher than that of clinical guidelines [23]. On the other hand, a substantial proportion of patients one year after transcatheter edge-to-edge repair techniques deteriorate independently on the degree of FMR proportionality at the baseline [24]. Whether the baseline assessment of proportionality in FMR patients referred for percutaneous annuloplasty with the Carillon device could predict long-term clinical effects remains to be established in larger clinical trials. Sub-analysis of the results of the currently ongoing EMPOWER trial may provide appropriate data.

## 5. Limitations

Our study has several limitations. First, it is a post hoc analysis of data from pooled clinical trials. The results should therefore be confirmed in additional prospective multicenter trials with a larger patient cohort. Second, the follow-up time was one year after the procedure, which might have been too short a time span to identify the impacts of other parameters for long-term CMCS results.

## 6. Conclusions

In FMR patients undergoing the Carillon procedure, higher baseline LV volumes may predict more pronounced reverse remodeling, defined as a decrease in LV volumes. Higher values of vena contracta may predict larger reductions in mitral annular areas. High baseline LV diameters may suggest improvements in LV volumes in disproportionate FMR and in LV diameters in proportionate FMR, probably due to different LV geometries.

## Figures and Tables

**Table 1:** Correlations between changes in parameters after 12 months (change) and baseline values (baseline) among implanted patients with disproportionate FMR (EROA/LVEDV > 0.15).

	RV Baseline	EROA Baseline	VC Baseline	LVEF Baseline	LVEDV Baseline	LVESV Baseline	LVEDD Baseline	LVESD Baseline	MAA Baseline
RV Change	−0.403 ^a^**	−0.274 ^a^*	−0.016 ^a^	0.197 ^a^	−0.149 ^a^	−0.178 ^a^	−0.085 ^a^	−0.080 ^a^	0.139 ^a^
EROA Change	−0.339 ^b^	−0.342 ^b^*	−0.205 ^b^	0.297 ^b^*	−0.263 ^a^	−0.284 ^a^*	−0.114 ^b^	−0.084 ^b^	0.126 ^b^
VC Change	0.097 ^a^	0.157 ^a^	−0.429 ^a^**	0.259 ^a^	−0.093 ^a^	−0.146 ^a^	−0.045 ^a^	−0.072 ^a^	0.069 ^a^
LVEF Change	−0.070 ^a^	−0.007 ^a^	0.152 ^a^	−0.297 ^a^*	−0.042 ^a^	0.050 ^a^	−0.102 ^a^	−0.082 ^a^	−0.123 ^a^
LVEDV Change	−0.291 ^a^*	−0.257 ^a^	−0.070 ^a^	0.394 ^a^**	−0.493 ^a^**	−0.487 ^a^**	−0.363 ^a^**	−0.532 ^a^**	−0.376 ^a^**
LVESV Change	−0.251 ^a^	−0.272 ^a^	−0.150 ^a^	0.483 ^a^**	−0.352 ^a^*	−0.408 ^a^**	−0.264 ^a^	−0.372 ^a^**	−0.197 ^a^
LVEDD change	0.112 ^a^	0.143 ^a^	−0.211 ^a^	0.225 ^a^	−0.131 ^a^	−0.182 ^a^	−0.226 ^a^	−0.195 ^a^	0.021 ^a^
LVESD Change	0.211 ^a^	0.252 ^a^	−0.152 ^a^	0.232 ^a^	−0.100 ^a^	−0.170 ^a^	−0.192 ^a^	−0.297 ^a^*	−0.013 ^a^
MAA Change	−0.057 ^a^	0.017 ^a^	−0.374 ^a^*	0.058 ^a^	0.016 ^a^	−0.014 ^a^	0.201 ^a^	0.179 ^a^	0.090 ^a^

^a^ Spearman’s rank correlation coefficient; ^b^ Pearson’s correlation coefficient. Text in bold indicates statistical significance, with significance levels of ** *p* < 0.01 and * *p* < 0.05. MR regurgitant volume (RV), effective regurgitant orifice area (EROA), vena contracta (VC), LV ejection fraction (LVEF), LV end-diastolic volume (LVEDV), LV end-systolic volume (LVESV), LV end-diastolic diameter (LVEDD), LV end-systolic diameter (LVESD), and mitral annulus area (MAA).

**Table 2:** Correlations between changes in parameters after 12 months (change) and baseline values (baseline) among implanted patients with proportionate FMR (EROA/LVEDV≤ 0.15).

	RV Baseline	EROA Baseline	VC Baseline	LVEF Baseline	LVEDV Baseline	LVESV Baseline	LVEDD Baseline	LVESD Baseline	MAA Baseline
RV Change	−0.233 ^a^	−0.132 ^b^	−0.421 ^b^	0.283 ^b^	0.029 ^b^	−0.128 ^b^	−0.246 ^b^	−0.064 ^b^	0.039 ^b^
EROA Change	−0.113 ^a^	−0.258 ^b^	−0.510 ^b^*	0.326 ^b^	−0.042 ^b^	−0.219 ^b^	−0.204 ^b^	−0.014 ^b^	0.088 ^b^
VC Change	−0.363 ^a^	−0.161 ^b^	−0.598 ^b^*	−0.170 ^b^	−0.157 ^b^	−0.138 ^b^	−0.227 ^b^	−0.045 ^b^	−0.205 ^b^
LVEF Change	0.030 ^a^	−0.006 ^b^	−0.068 ^b^	−0.375 ^b^	−0.116 ^b^	0.066 ^b^	0.224 ^b^	0.126 ^b^	−0.126 ^b^
LVEDV Change	−0.334 ^a^	−0.423 ^b^	0.376 ^b^	0.221 ^b^	−0.459 ^b^*	−0.496 ^b^*	−0.178 ^b^	−0.202 ^b^	−0.436 ^b^
LVESV Change	−0.259 ^a^	−0.298 ^b^	0.240 ^b^	0.458 ^b^*	−0.276 ^b^	−0.450 ^b^*	−0.298 ^b^	−0.273 ^b^	−0.215 ^b^
LVEDD Change	−0.181 ^a^	−0.093 ^b^	−0.015 ^b^	0.156 ^b^	−0.302 ^b^	−0.326 ^b^	−0.662 ^b^**	−0.469 ^b^*	−0.142 ^b^
LVESD Change	−0.234 ^a^	−0.037 ^b^	0.121 ^b^	−0.059 ^b^	−0.270 ^b^	−0.232 ^b^	−0.511 ^b^*	−0.572 ^b^**	0.009 ^b^
MAA Change	−0.050 ^a^	0.108 ^b^	−0.459 ^b^*	0.328 ^b^	0.133 ^b^	−0.034 ^b^	0.190 ^b^	−0.010 ^b^	0.011 ^b^

^a^ Spearman’s rank correlation coefficient; ^b^ Pearson’s correlation coefficient. Text in bold indicates statistical significance, with significance levels of ** *p* < 0.01 and * *p* < 0.05. MR regurgitant volume (RV), effective regurgitant orifice area (EROA), vena contracta (VC), LV ejection fraction (LVEF), LV end-diastolic volume (LVEDV), LV end-systolic volume (LVESV), LV end-diastolic diameter (LVEDD), LV end-systolic diameter (LVESD), and mitral annulus area (MAA).

## Data Availability

The original contributions presented in this study are included in the article. Further inquiries can be directed to the corresponding author.
